# The unusual case of the irretrievable colonoscope

**DOI:** 10.1002/ccr3.1211

**Published:** 2017-10-23

**Authors:** Joshua Hammerschlag, Travis Ackerman, Brian Hodgkins

**Affiliations:** ^1^ Colorectal Surgery Monash Health Melbourne Victoria Australia

**Keywords:** Colonoscopy, colorectal surgery, foreign body, gastroenterology

## Abstract

It is important that invasive procedures are rationalized, even relatively safe ones like colonoscopy, to minimize the risk of harm to patients.

## Background

Colonoscopy is a common and safe procedure. Nevertheless, there are a number of patients who suffer minor complaints and a very small percentage of patients in whom serious complications occur. We report a case of an elective colonoscopy being complicated by not being able to retrieve the colonoscope, a complication which to our knowledge has not yet been reported in the literature.

## Case presentation

A previously well 72‐year‐old female presented to a tertiary hospital for an elective colonoscopy. The indication was anemia, intermittent abdominal pain, and recently altered bowel habits. The patient had had several colonoscopies in the past although not for a number of years. Her previous colonoscopies were noted as being difficult and on the most recent attempt, the endoscopist failed to reach the cecum. The difficulties encountered in those procedures were attributed to significant diverticulosis.

In anticipation of a challenging colonoscopy, a pediatric colonoscope was used. Per rectum exam was unremarkable, and the bowel prep was described as average. After some initial difficulties, the endoscopist had some success advancing the scope using water irrigation to distend the bowel. However, due to significant looping, the scope could not be advanced past the splenic flexure. At this point, the endoscopist encountered resistance and was unable to retrieve the colonoscope. Fluoroscopy was performed, and a tight angulation in the sigmoid colon was observed.

The patient was given a general anesthesia and paralyzed in an attempt to retrieve the colonoscope. However, this too was unsuccessful. In conjunction with the on‐call colorectal surgeon, a decision was made to transfer the patient to the operating theater for an exploratory laparotomy. Intraoperatively, significant intraabdominal adhesions were encountered, and a large phlegmonous mass was noted abutting and constricting the patient's sigmoid colon. It became apparent that the adhesion between the patient's sigmoid colon and this inflammatory mass were responsible for the unusual predicament. The scope had looped in the sigmoid colon, and both the afferent and efferent limbs were fixed at the same level. Furthermore, the loop had twisted in a volvulus‐type configuration. This meant that as the surgeon tried to either withdraw or advance the scope, the whole loop would move against the fixed adhesions, and the scope would not progress. The surgeon performed a limited adhesiolysis in order to free the sigmoid colon from the inflammatory mass. The colonoscope was then finally able to be retrieved. On withdrawing the colonoscope, the surgeon carefully examined the mucosal wall for signs of any injury, but none were noted.

The patient was carefully monitored on the ward and discharged four days later. The patient has been reviewed as an outpatient and has recovered well from the procedure.

## Discussion

Colonoscopy is a common procedure employed for both diagnostic and therapeutic purposes. It is the gold standard investigation and treatment modality for a wide range of gastrointestinal pathologies including both benign and malignant conditions. While generally safe, there are a significant number of patients who suffer minor complaints, including bloating and abdominal pain postprocedure. Although uncommon, there are also some serious complications that may occur. Significant bleeding and perforation are the most prevalent, but even so, the incidences are thought to be as low as 0.1%–0.3% 1. A number of novel complications have been reported in the literature 2, but none have involved being unable to retrieve the colonoscope.

This case presented the surgeon and endoscopist with multiple potential problems; one of which was that the colonoscopy was in effect a rectal foreign body. This carries its own risks of mucosal damage or inadvertent bowel perforation 3. Another potential problem was the potential risk of splenic injury. There are documented cases of splenic injury caused by difficulties in intubation, looping or traction on the either splenocolic ligament or adhesions to this ligament 4. An overaggressive attempt to retrieve the colonoscope in this case could quite easily have caused significant injury to the patient's spleen.

A compounding factor during the procedure that was noted was inadequate bowel preparation. Inadequate bowel preparation for colonoscopy can result in missed lesions, canceled procedures, increased procedural time, increased costs, and a potential increase in adverse event rates 5, 6. It was known that the colonoscopy for this patient was going to be difficult owing to severe diverticular disease 7 and as such everything should have been done to improve the chances of success. Although not directly responsible for the colonoscope becoming stuck, the inadequate bowel preparation in this case did make the procedure more difficult.

Finally, the most important factor in avoiding complications from colonoscopy may be a more critical rationalization of whom should undergo the procedure. This was an unfortunate complication that occurred despite reasonable preoperative preparation by the endoscopy team which anticipated the difficulties faced due to the patient's previous difficult colonoscopies. In hindsight, given the patient's history perhaps an alternative means of evaluation of the colon such as CT colonography may have been a more suitable means by which to investigate this patient.

## Conclusion

Colonoscopy is a safe investigation that remains the most effective way to diagnose and treat a range of gastrointestinal pathologies. Complications are reportedly rare but potentially serious. We describe a novel complication of a commonly performed procedure which to our knowledge has never before been described in the literature. It is important that invasive procedures are rationalized, even safe ones to minimize risks of harm to our patients.

## Authorship List

Dr. JH: corresponding author, complied workings and formulated manuscript; Mr. TA: Background research and prepared foundation for case report; Mr. BH: Operating surgeon, provided edits and guidance.

## Conflicts of Interest

None declared
